# Identification of extremely hard coke generation by low-temperature reaction on tungsten catalysts via *Operando* and in situ techniques

**DOI:** 10.1038/s41598-021-86949-x

**Published:** 2021-04-13

**Authors:** Thotsatham Takkawatakarn, Supareak Praserthdam, Sippakorn Wannakao, Joongjai Panpranot, Piyasan Praserthdam

**Affiliations:** 1 Center of Excellence On Catalysis and Catalytic Reaction Engineering, Department of Chemical Engineering, Faculty of Engineering, Chulalongkorn University, Bangkok, 10330 Thailand; 2 SCG Chemicals Co., Ltd., 1 Siam Cement Road, Bangsue, Bangkok, 10800 Thailand

**Keywords:** Chemical engineering, Catalysis

## Abstract

The coke formation in the catalytic system mainly cause to the catalyst deactivate resulting the dramatic decreasing of the catalyst performance then the catalyst regeneration was required. In this study, adding MgO physically mixed with WO_3_/SiO_2_ catalysts were prepared and compared with the ones prepared by physically mixing with SiO_2_. Adding MgO affected the generation of new species of coke deposited on WO_3_/SiO_2_ and MgO itself. Comparing the reaction temperature when adding MgO between at 300 and 450 °C, the different pathway of reaction and the coke formation were found. At 450 °C, the metathesis reaction was more pronounced and the lower temperature of coke deposited on WO_x_/SiO_2_ was found. Surprisingly, the extremely hard coke occurred during reaction at 300 °C that the maxima of coke formation was found over 635 °C. This due to the fact that the reduction of reaction temperature from 450 to 300 °C affected the decreasing of the metathesis activity. Conversely, the increasing of dimerization and isomerization of butenes-isomer was observed especially 1-butene and iso-butene. Thus, it could suggest that those quantity of them play the important role to generate the charged monoenyl or cyclopentenyl species by participating with ethene through the dimerization, resulting in the formation of extremely hard coke.

## Introduction

Industrially, tungsten metal based catalysts are used in various reactions. One of the most important reaction is olefin metathesis reaction^1–6^. Metathesis reaction are used to transform the olefin in double bond position to produce the olefin products that have more value in the petrochemical industries^7^. Commercially, ethylene can react with propylene via metathesis reaction to enhance propylene production^8^. However, isomerization of 2-butene as side reaction can occur with metathesis reaction. When 2-butene was isomerized to 1-butene or iso-butene during the metathesis reaction take place, they can pronounce to generate long chain of olefin hydrocarbon like pentene or hexene in the system by secondary metathesis reaction. Long chain olefins caused to coke precursor generation and finally they converted to the coke formation depositing on the surface of the catalyst that mainly affect catalytic performance^8^. The coke formation in the catalytic system mainly cause to deactivate the performance and activity of catalysts^8,9^. When the high temperature of coke was generated, its necessary to increase the regenerated temperature that induced the metal sintering^10^.The alternative way to prevent these phenomena are using the catalysts that can convert the 1-butene or iso-butene back to the 2-butene by isomerization. Magnesium oxide (MgO) is the most important catalyst that use to catalyze the isomerization reaction^11,12^. Commercially, MgO catalysts may be mixed with metathesis catalysts which originally existed only metathesis catalyst for performing both double-bond isomerization and metathesis in the same reactor^13^. No matter if MgO physically mixed or put it on the top of metathesis catalyst in reactor. Beneficially, it help to prevent the coke formation and reduce the consumption of coke deposited on the surface catalyst, on the other hand it changed some phenomena of reaction pathway on the catalyst such as generated soft coke and reduced the hard coke^14^. It is the interesting point to deep study and understanding about the phenomena that affect to the catalytic activity. *Operando* ultraviolet–visible light spectroscopy (*Operando* UV–vis) is used to characterize and observe the species of coke that deposited on the catalyst surface during reaction^15–17^. Using this technique could monitored the occurrence of coke at real time on the surface of the catalyst while the reaction is occurred.

Acidity plays an important role on the metathesis activity of WO_x_/SiO_2_ catalysts^1,18,19^. Ammonia was used as probe molecule to adsorb on the catalyst then in situ DRIFTS technique is used to classify type of acid sites which are Brønsted and Lewis acid sites^20,21^. With ammonia chemisorption technique led to understand the correlation of types of acidity and catalytic activity of fresh WO_x_/SiO_2_ catalysts. Moreover, acidity is the parameter that directly effect to the adsorption of reactants. Many studies have shown that the adsorption strength of the substance is related to the amount of acid on the catalyst. Therefore, in this research, we try to develop techniques to analyze the adsorption strength of different catalysts by using in situ DSC combining with in situ DRIFTS^22^.

The present study, we attempt to develop the *Operando* UV–vis technique by installing multiple probes to measure the coke formation with the different height of the catalyst packed bed in reactor. Noteworthy, inhomogeneity of coke formation was inhomogeneous axial deactivation along the packed bed of catalyst. Therefore, when combined the *Operando* results of multi-UV–vis probes with the composition of different substances that measured by GC-FID, it is possible to find some correlation of various parameter that effect to the catalytic activity and coke formation.

## Methods

### Preparation of yWO_x_/SiO_2_ catalysts

WO_x_/SiO_2_ catalysts with 5 and 9%wt. of tungsten metal loading s were prepared by the incipient wetness impregnation of amorphous silica gel (High-purity grade, 35–60 mesh, pore size 150 Å supplied by Sigma-Aldrich) with an aqueous solution containing the desired amount of ammonium metatungstate hydrate ((NH_4_)_6_H_2_W_12_O_40_·xH_2_O, 99.9%, supplied by Sigma-Aldrich) as the tungsten precursor. The impregnated catalyst was dried for 2 h in ambient air and subsequently in an oven at 110 °C for 24 h, following by calcination in air higher than 500 °C to improve the stability of tungsten catalysts. There samples are labelled as 5WO_x_/SiO_2_ and 9WO_x_/SiO_2_.

### Preparation of MgO catalysts

Magnesium oxide was a commercial grade (specific surface area 167 m^2^/g), in the form of hollow with 4 × 5 mm extrudates which have been crushed and sieved to obtain the mesh size between 25 and 30 then was calcined at in air at temperature higher than 550 °C to remove the impurity.

The prepared catalysts of yWO_x_/SiO_2_ with different tungsten metal loadings were physically mixed with silica gel and magnesium oxide in a weight ratio of 1:1. They can be divided in two groups. First, the yWO_x_/SiO_2_ catalysts were physically mixed with the silica gel and were labelled as 5WO_x_/SiO_2_ + SiO_2_ and 9WO_x_/SiO_2_ + SiO_2_. Secondary, the yWO_x_/SiO_2_ catalysts were physically mixed with the magnesium oxide and were labelled as 5WO_x_/SiO_2_ + MgO and 9WO_x_/SiO_2_ + MgO.

### Catalyst characterization

**Temperature–programmed oxidation (TPO)** measurements were used to analyze the carbon that deposited on the spent catalyst. 0.30 g of each catalyst was placed in quartz tube under the 1% O_2_/He flow and heated with the heat rate 5 °C/min from 25 °C to 950 °C. In case of MgO to prevent the CO_2_ capture ability, it was pretreated to remove the CO_2_ in He to 700 °C before started the temperature-programed oxidation to measuring the amount of coke. Oxygen consumption and formation of reaction product as CO_2_ were monitored by a shimadzu gas chromatograph (GC-2014) equipped with TCD.

**Operando UV–vis spectroscopy coupled with GC-FID** measurements were performed using 0.8 g of catalyst in a customized quartz with rectangular fixed-bed reactor (ID = 40 mm × 10 mm), weight hourly space velocity (WHSV) of 2.1 h^−1^. Catalysts were packed in reactor and pretreated with 90 ml/min of N_2_ before reaction testing. A reactant of 4% ethylene and 2% *trans*-2-butene balanced in N_2_ was flowed through the catalyst at the difference reaction temperature 300 and 450 °C to monitor the coke formation during reaction. *Operando* UV–vis spectra were corrected every 1 min in reflection mode at top and bottom bed of the catalyst packed bed by using AvaSpec 2048L spectrometer connected to a high-temperature UV–vis optical fiber probe with fiber optic wires. For reaction measurement of coke generated with different probe molecule of *trans*-2-butene, 1-butene, and isobutene that contain 4% of olefin balanced in N_2_, the physically mixed of MgO and 9WO_x_/SiO_2_ were packed in to the quartz reactor that using the same pretreatment condition and test the reaction at 300 °C.

**In situ DRIFTS** (diffuse reflectance infrared Fourier transform spectroscopy) measurements were determined on the sample holder cup that the probe molecule can flow through the samples. The Praying Mantis High Temperature Reaction Chambers, diffuse reflection accessory from Harrick Scientific Product Inc., with two Potassium Bromide (KBr) windows and one SiO_2_ observation window on the chamber was used in conjunction with FT-IR from Bruker (vertex-70 spectrometer with a mercury cadmium telluride (MCT) detector kept at − 196 °C by liquid N_2_ during measurement). Prior to measurement, the fresh and spent catalysts were studied according to the following procedure: (1) the samples were preheated at 500 °C for 1 h in N_2_ (10 ml/min^−1^) with a heating rate of 10 °C/min under atmospheric pressure; (2) after pretreatment, the samples were cooled down to 50 °C and IR spectrum was record as the background spectrum; (3) subsequently, the probe molecule (NH_3_, ethylene, *trans*-2-butene, 1-butene, and iso-butene) were admitted through the sample until equilibrium adsorption. After purging the physisorbed probe molecules by N_2_ flow for 30 min, the FTIR spectra of adsorbed species on catalysts were collected simultaneously. Each spectrum was collected by averaging 128 scans with a resolution 2 cm^−1^ in the 4200–600 cm^−1^ range. All IR spectra of the adsorbed species were obtained by subtracting the background spectrum by using the OPUS software package (OPUS 7.5, Bruker Optik GmbH 2014).

**In situ DSC** (difference scanning calorimetry) measurements were carried out using C-600 Calvet Calorimetry, SETARAM Instrumentation to characterize the adsorption energy of difference probe molecule by pulse chemisorption techniques. The 200 mg of fresh and spent catalyst of WO_x_/SiO_2_ catalysts were loaded to gas circulation cell and installed into the furnace that cover with perfect insulation. The energy consumption was studied according to the following procedure: (1) the samples were pretreated before measurement at 500 °C under N2 atmospheric pressure; (2) after pretreatment, the samples were cooled down to the 50 °C; (3) After the output signal was stabled, the difference probe molecule were pulse fed to the sample and measured the total heat evolved by high sensitivity of 3D Calvet sensor at 30 µW/mW and 0.10 µW of resolution. All the results of total energy were calculated and converted by CALISTO software.

All the characterization techniques of experimental devices were shown by the following steps.

Before testing the catalytic activities.

- Measuring the type of acidity by in situ DRIFTS techniques (probe molecules: ammonia).
- Measuring the adsorption energy by in situ DSC (fresh catalysts).

Catalytic activities testing.

- Real time monitoring the surface reaction by Operando UV–vis spectroscopy coupled with GC-FID.

After testing the catalytic activities.

- Measuring the adsorption energy by in situ DSC (spent catalysts to compared with the fresh catalysts).
- Measuring the species and quantities of coke formation on the spent catalyst by TPO techniques.

## Results and discussion

### In situ DRIFTS with NH_3_ adsorption technique

In order to understand the respective role of Lewis and Brönsted acidic sites in studied metathesis catalysts, in situ DRIFTS with NH_3_ adsorption technique was carried out and the results are shown in Table 1. The quantity and species of Lewis and Brønsted acid sites can be divided into four groups that related to the positive adsorption bands of NH_4_^+^ bending vibrations. The positive adsorption bands were found at 1680, 1621, 1472, and 1280 cm^−1^^15,21,23,24^. The former assigned the bending vibration of NH_4_^+^ species at 1472 cm^−1^ and 1680 cm^−1^ resulting adsorbed of NH_3_ on Si–OH and W–OH groups on the position of Brønsted acid site, while N–H bands bending vibration of molecularly adsorbed of NH_3_ at 1280 cm^−1^ and 1621 cm^−1^ were attributed to Lewis acid sites^20^. Considering the DRIFTS spectra of the WO_x_/SiO_2_ catalyst with different tungsten metal loading, the Brønsted acid sites at 1472 cm^−1^ (Brønsted type I), Lewis acid site at 1280 cm^−1^ (Lewis type I) and 1621 cm^−1^ (Lewis type II) were generated at 9% of tungsten metal loading that caused of the agglomeration of tungsten metal oxide on the silica supported, correlating well with the literatures^25–27^. However the Brønsted acid site at 1680 cm^−1^ (Brønsted type II ) decreased when increasing tungsten metal loading^22^.

**Table 1:** Quantitave and qualitative of BrØnsted and Lewis acid site results by in situ DRIFTS of ammonia chemisorption^a^.

Catalysts	BrØnsted acid (a.u.)	Lewis acid (a.u.)	Bronsted acid (a.u.)	Lewis acid (a.u.)
	(1680 cm^−1^); Type II	(1621 cm^−1^); Type II	(1472 cm^−1^); Type I	(1280 cm^−1^); Type I
SiO_2_	0.162	0.302	0.024	0.041
5WO_x_/SiO_2_	0.071	0.408	0.708	0.198
9WO_x_/SiO_2_	0.022	0.719	1.528	0.386

^a^Ammonia adsorption on the DRIFTS cell at 50 °C, 0.1 Mpa (after pretreated in N_2_; 1 h 550 °C).

### Catalytic analysis and general aspects of Metathesis reaction

**Figure 1:**
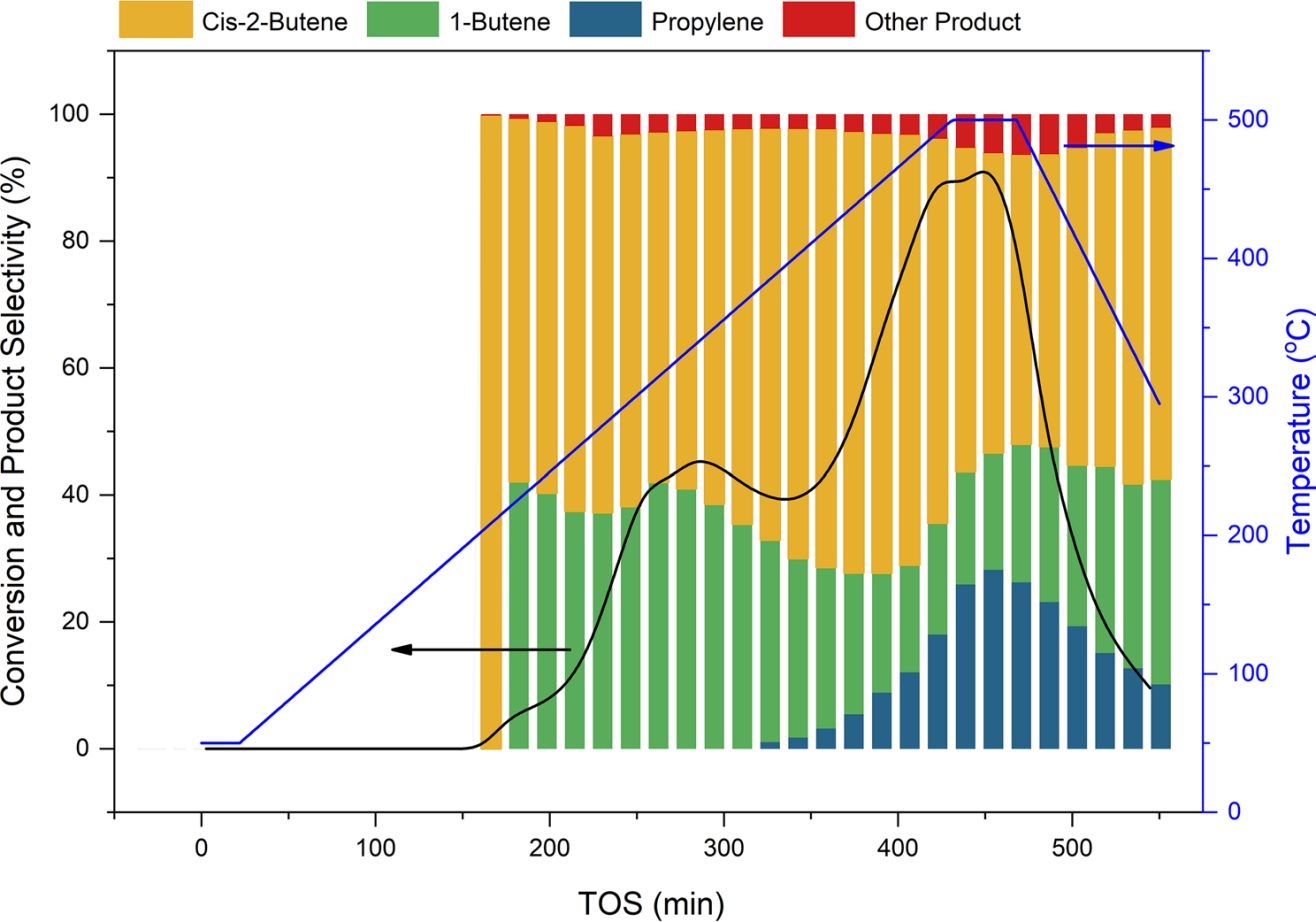
Difference reaction pathways on 9WO_x_/SiO_2_. The temperature programmed from 50 to 500 °C for reaction testing to observe the depending reaction pathways. The experiment was tested with 4% ethene and 2% *trans*-2-butene on 9WO_x_/SiO_2_ catalyst (WHSV = 0.21 h^−1^; P = 0.1 MPa).

The 9WO_x_/SiO_2_ catalyst was firstly considered to test the reaction and monitor the possibility of different reaction pathways with 4% ethene and 2% *trans*-2-butene as the reactants. The experiment was designed to use the temperature programing from 50 to 500 °C with heating rate 1 °C/min. The composition of substance was detected by GC-FID. The conversion and selectivity profiles as a function of reaction time are shown in Fig. 1. When temperature raised up to 180 °C, the isomerization reaction was starting occurred and the mainly products were *cis*-2-butene and 1-butene. After the temperature programing increase to 200 °C, iso-butene and some of C^5+^ products were generated in the catalytic system cause to dimerization reaction. Then metathesis as the main reaction was occurred when the temperature increase to 320 °C which consistent with the previous research of olefin metathesis on tungsten metal oxide that required high temperature for catalytic reaction^14,25^.

From the reaction pathways on tungsten silica supported catalyst function to the temperature, we decided to study the reaction temperature at 300 °C and 450 °C. As mentioned above at 300 °C was not functioned to the occurrence of the metathesis reaction, which was an advantage that allowed us to study in the framework of isomerization and dimerization (the initial reaction of oligomerization). Then, adding MgO to the catalytic system, it is easier to demonstrate the phenomena that occurred on both of isomerization on MgO and WO_x_/SiO_2_ catalysts. Thus, for monitoring the effect of adding MgO, reaction testing of yWO_x_/SiO_2_ have been divided to two groups. First, as silica gel is barely acidic, yWO_x_/SiO_2_ was physically mixed with silica gel to employ for comparison purposes. The conversion and selectivity obtained on the various catalysts after 5 h reaction time are summarized in Table 2. Comparison of the difference tungsten metal loading at reaction temperature 450 °C indicated that increasing of tungsten metal loading affect to the increasing of the *cis*-2-butene and C_5_^+^ products in the reaction system due to the more pronounced of acidity on the catalyst (Table 1). Correlating with the acidity and the activity, the improvement of metathesis reaction was increase with increasing of acidity on WO_x_/SiO_2_. However, it also affected to increase the isomerization of *trans*-2-butene followed with secondary metathesis reaction to generate C_5_^+^ products.

**Table 2:** Conversion and product selectivity of SiO_2_, MgO, WO_x_/SiO_2_ + SiO_2_ and WO_x_/SiO_2_ + MgO catalysts of reaction temperature at 300° and 450 °C (WHSV = 0.21 h^−1^; P = 0.1 Mpa)^a^.

Catalysts	Reaction temperature (°C)	Conversion (%)^b^	Selectivity (%)					
			Propene	1-Butene	iso-Butene	*Cis*-2-Butene	2-Pentene	C_6_^=^, C_6_^=+^
SiO_2_	300	12.1	0.18	59.44	–	39.84	0.54	–
MgO		28.1	0.11	49.38	1.59	48.65	0.27	–
5WO_x_/SiO_2_ + SiO_2_		21.5	0.18	40.31	0.30	58.97	0.16	0.08
9WO_x_/SiO_2_ + SiO_2_		28.9	0.21	39.03	0.81	59.75	0.11	0.09
5WO_x_/SiO_2_ + MgO		39.2	1.08	37.21	0.76	60.54	0.28	0.13
9WO_x_/SiO_2_ + MgO		41.1	1.81	36.81	1.12	59.34	0.72	0.20
SiO_2_	450	15.2	0.35	41.08	–	56.70	1.87	–
MgO		58.5	0.12	45.12	2.01	51.19	1.56	0.00
5WO_x_/SiO_2_ + SiO_2_		32.2	7.83	52.81	0.89	36.24	2.09	0.14
9WO_x_/SiO_2_ + SiO_2_		47.8	10.01	44.09	1.20	41.51	2.94	0.25
5WO_x_/SiO_2_ + MgO		60.1	10.69	46.26	1.14	39.51	2.20	0.20
9WO_x_/SiO_2_ + MgO		63.8	11.12	39.53	1.32	44.42	3.19	0.42

^a^WHSV; weight of reactants were calculated from mixed reactant of 2% *trans*-2-butene and 4% ethene balanced in N_2_ (P = 0.1 Mpa).

^b^Conversion were calculated base on *trans*-2-butene consumption.

Second, yWO_x_/SiO_2_ was physically mixed with MgO and the reaction was tested at 450 °C. It is clearly seen that the admixed MgO with the yWO_x_/SiO_2_. resulted in much higher conversion of *trans*-2-butene compared to the one physically mixed with silica gel due to its isomerization function. It is noticed that when comparing the isomerization sites on yWO_x_/SiO_2_ with MgO, the isomerization sites on the yWO_x_/SiO_2_ were not strong enough to convert 1-butene and *cis*-2-butene to *trans*-2-butene. Therefore, the increasing of isomerization active species was beneficially helped to increase opportunities of *trans*-2-butene converted to 1-butene. On the other hand, MgO could convert 1-butene back to 2-butene and increase the possibility of propene production. When considering from pathway of reaction, 1-butene can react with 2-butene and other 1-butene to form the C_5_^+^ by secondary metathesis reaction. Thus, occurring of secondary metathesis affected the additional propylene in the reaction system as shown in Table 2. For reaction testing at 300 °C, it was consistent to the catalytic activity at 450 °C as admixed MgO in the catalyst system provoked the conversion and propylene selectivity.

### Characterization of coke formation on catalysts

**Figure 2:**
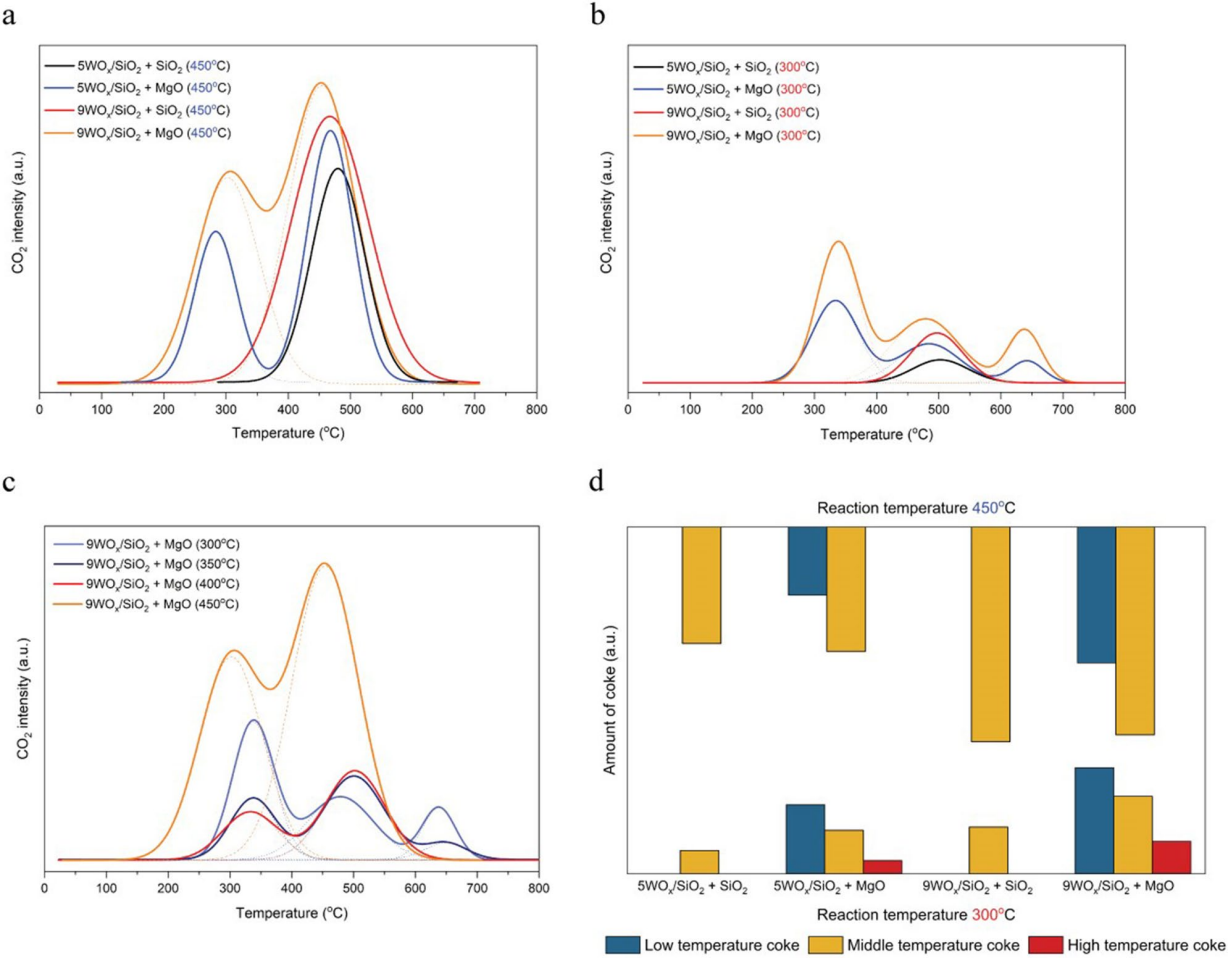
Temperature programmed oxidation. The results of spent catalysts with different tungsten metal loading and physically mixed with SiO_2_ and MgO after 5 h of reaction. (**a**) The TPO of reaction testing at temperature 450 °C. (**b**) The TPO of reaction testing at temperature 300 °C. (**c**) The TPO of 9WO_x_/SiO_2_ + MgO at the various reaction temperature from 300–450 °C to monitoring the extremely hard coke generated depending on reaction pathways. (**d**) The quantitative comparison of coke that deposited on the catalysts that shown the low, middle, and high temperature coke.

The carbon deposit on surface of both yWO_x_/SiO_2_ and MgO catalyst was investigated by temperature-programmed oxidation (TPO). The CO_2_ profiles are illustrated in Fig. 2. For observing the coke formation on each catalyst, yWO_x_/SiO_2_ and MgO were prepared with different particle size that could easier to separate by sieving. From the literature, yWO_x_/SiO_2_ catalyst had one species of coke formation around temperature of 450–550 °C. Coke formation mostly deposited on yWO_x_/SiO_2_ catalyst meanwhile there was a slightly deposited on MgO^14^. As can be seen in Fig. 2a, coke formation on yWO_x_/SiO_2_ catalyst physical mixed with MgO had two species when reaction was tested at 450 °C. One was the main coke formation at around 450–550 °C as the same pattern like the system of yWO_x_/SiO_2_ + SiO_2_ catalyst. However, there was the lower peak at temperature around 280–340 °C, represented to soft coke formation. From the above discussion showed the ability of MgO could catalyze isomerization reaction that cause of the increasing of C_5_^+^ product in the reaction system. Correlating the reaction pathway and coke formation, it can be concluded that when the reaction system comprised with higher molecule weight of olefin, its provoked the opportunity of coke formation on the catalyst. Comparing between 5WO_x_/SiO_2_ and 9WO_x_/SiO_2,_ both catalysts comprised with the same species of coke depositing. Indeed, higher tungsten metal loading provoked coke formation due to higher acidity and higher conversion. Accordingly, coke content was related proportionally with the activity.

It was interesting that when the reaction was tested at 300 °C, three maxima peaks were observed. The additional species of coke formation at temperature oxidation around 645 °C was found on the spent yWO_x_/SiO_2_ + MgO catalyst (Fig. 2b). It was clear that the extreme hard coke formation preferable occurred at lower temperature of reaction (300 °C) when adding MgO physical mixed yWO_x_/SiO_2,_ Accordingly, the increasing of reaction temperature from 300 to 450 °C induced to decrease the carbon deposited on temperature oxidation at 645 °C and disappeared after increase the reaction temperature over 400 °C as can be seen in Fig. 2c and the quantitative comparison of hard coke can be seen in Fig. 2d.

As discussed above at 300 °C of reaction, only isomerization and dimerization reaction on both site of yWO_x_/SiO_2_ and MgO were occurred. Thus, to investigate the phenomena of soft coke formation, the selected reaction temperature was chosen at 300 °C due to disposing of metathesis function on yWO_x_/SiO_2_ in the system. Since is reported in literature that the dimerization reaction was played a greater role to generate the C_5_^+^ from butene itself or co-dimerization of ethene^28,29^, This is in accordance with our results which can be seen from the increasing of C_5_^+^ product but did not produced more the quantities of propene as in case of testing the reaction at 450 °C.

**Figure 3:**
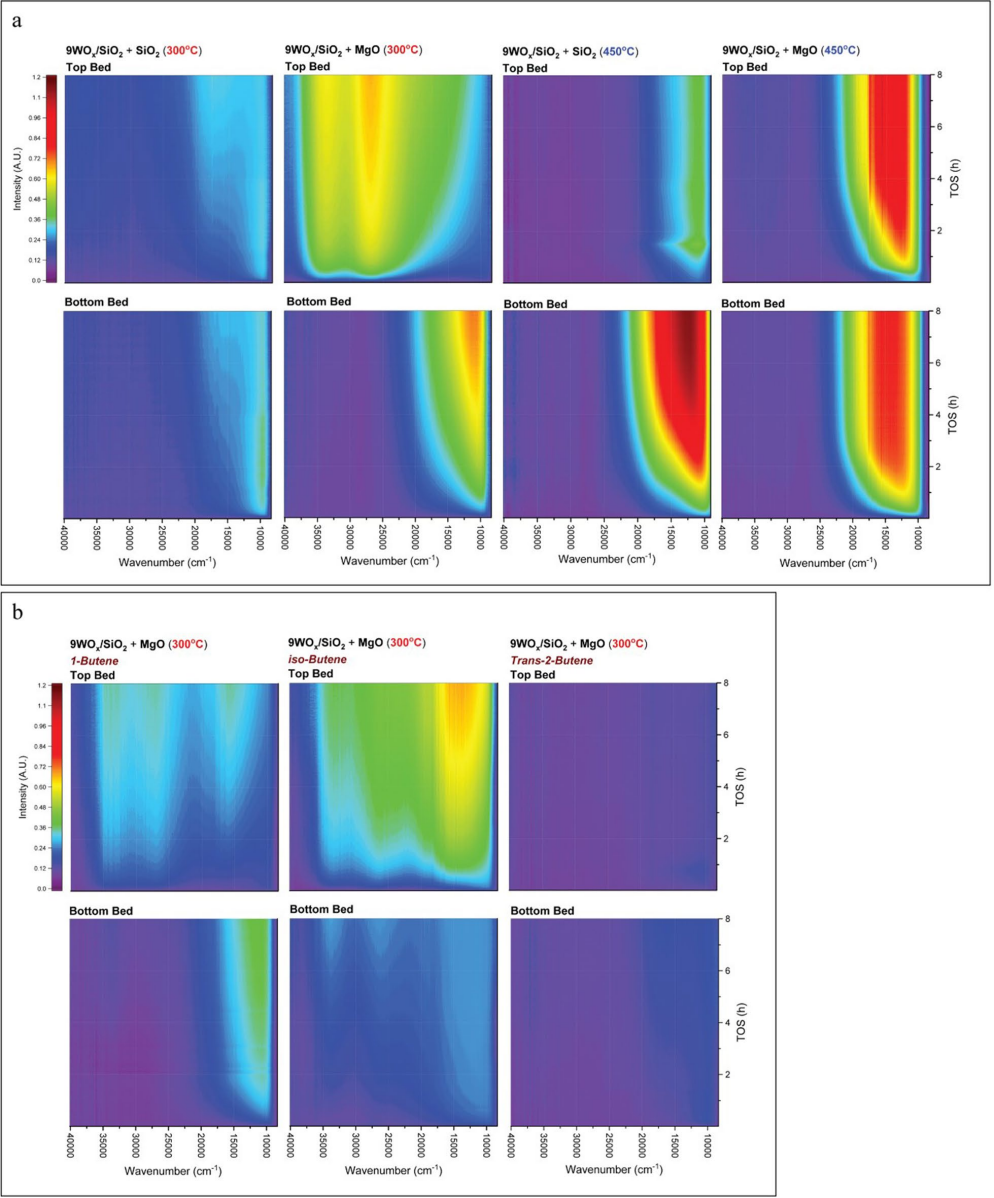
*Operando* UV–vis spectroscopy with multi probes. The *Operando* spectrum results of top and bottom of catalytic packed bed. (**a**) The 9WO_x_/SiO_2_ + SiO_2_ and 9WO_x_/SiO_2_ + MgO with different reaction temperature at 300 and 450 °C. (**b**) The spectrum results of reaction testing with various reactant as 1-butene, iso-butene, and *trans*-2-butene which 4% of alkene balanced in N_2_.

In order to further understanding about the coke formation on studied catalysts along with the height of the catalyst packed bed, *Operando* UV–vis was carried out and the results are shown in Fig. 3. The spectrum band around wavenumber 12,500–23,000 cm^−1^ represented to charged and neutral poly-aromatics. And the spectrum around wavenumber 23,000–27,000 cm^−1^ was assigned to the charged poly-alkylated benzene and charged alkylated naphthalenes. Meanwhile the spectrum at 30,000–35,000 cm^−1^ assigned to the charged monoenyl/cyclopentenyl species^15,16^. Coke formation was monitored at the top bed of 9WO_x_/SiO_2_ + MgO during reaction at 300 °C and the results showed that the high quantity of the charged of poly-alkylated benzene and monoenyl/cyclopentenyl species was occurred^16^ (Fig. 3a). In conversely, the spectrum band charged and neutral poly-aromatic slight increase after 4 h on stream. It was interesting that at bottom bed was generated the different pattern of coke formation. As the metathesis reaction was not occurred at this temperature, thus the occurrence of the high quantity of the charged of poly-alkylated benzene and monoenyl/cyclopentenyl species cause of the role of isomerization and dimerization. These attributed the extreme hard coke formation on the surface catalyst that revealed by TPO results. When increasing the reaction temperature to 450 °C, the spectrum band around wavenumber 12,500–23,000 cm^−1^ that represented to charged and neutral poly-aromatics were slight increase from top bed then considerable increased at bottom bed which can observed from the spectrum intensity (Fig. 3a)^15–17^.

Furthermore, we have to further investigate the other parameters such as kind of C4-feed stream that affected to coke formation on the WO_x_/SiO_2_ + MgO catalyst. Thus, we try to find that correlation by using the isomer of C4-feed stream as the reactant including *trans*-2-butene, 1-butene, and iso-butene. Each C4-alkene balanced with N_2_ was fed into the reactor to observe the coking pattern with *Operando* UV–vis (Fig. 3b). Combining the results from Fig. 3a,b 1-butene and iso-butene as the reactant showed the same pattern with the 4% ethene and 2% *trans*-2-butene. Iso-butene were reacted and generated coke faster than 1-butene. Meanwhile 1-butene generated the charged and neutral poly-aromatics slight increase on the top bed of catalyst then affected the movement of coke precursors to the bottom bed and generated the coke formation at wavenumber around 10,000 to 15,000 cm^−1^. Interestingly, when *trans*-2-butene, was fed, the pattern of coke formation was difference from those ones, coke accumulation was not occurred on the along of catalyst pecked bed. These contributed that the catalytic system that fed only *trans*-2-butene, without ethene interaction it’s almost impossible to generate the coke at 300 °C (Fig. 3b).

The strength of adsorption that combining between in situ DSC and DRIFTS was calculated from the ratio between the adsorption energy and amount of pulsed chemisorption of the probe molecules on fresh and spent catalysts. This value could suggested the ability of difference substances that adsorb on the catalyst surface after the reaction was occurred (Table 3)^22^. The adsorption ability of reactant and product trended to increase, especially using iso-butene as the probe molecule. At reaction temperature 300 °C, yWO_x_/SiO_2_ + MgO catalyst was noticeable increase of strength of adsorption which consistent with *operando* UV–vis results.

**Table 3:** Strenght of adsorption with the various probe molecules that adsorbed on the WO_x_/SiO_2_ + SiO_2_ and WO_x_/SiO_2_ + MgO catalysts.

Catalysts^a^		Reaction Temperature (°C)	Strength of Adsorption^b^			
First	Second		Ethene	*trans*-2-Butene	1-Butene	iso-Butene
**Fresh catalyst**						
5WO_x_/SiO_2_	SiO_2_	–	1.35	2.01	1.71	2.01
5WO_x_/SiO_2_	MgO		1.42	2.75	2.44	2.67
9WO_x_/SiO_2_	SiO_2_		0.98	1.53	2.43	1.68
9WO_x_/SiO_2_	MgO		1.06	1.94	2.94	1.87
Spent catalyst*						
5WO_x_/SiO_2_	SiO_2_	300	1.87	2.20	1.62	3.22
5WO_x_/SiO_2_	MgO		2.74	2.68	2.53	4.21
9WO_x_/SiO_2_	SiO_2_		1.77	1.97	2.38	2.83
9WO_x_/SiO_2_	MgO		3.06	2.64	3.08	4.29
5WO_x_/SiO_2_	SiO_2_	450	1.56	1.86	2.41	2.76
5WO_x_/SiO_2_	MgO		1.68	2.16	2.78	3.02
9WO_x_/SiO_2_	SiO_2_		1.03	1.91	2.66	3.11
9WO_x_/SiO_2_	MgO		1.17	2.31	3.14	3.57

Compared between fresh and spent catalysts with different of reaction temperature.

^a^First and second catalysts were mixed by physically process.

^b^Strenght of adsorption; Ratios of adsorption energy (J·g catalyst^−1^) from in situ DSC measurements and DRIFTS Area (a.u. ·g catalyst^−1^) (area integrated at wavenumber 1660–1720 cm^−1^; isolated *C*=*C* and *C*=*C* conjugated with aryl) from in situ DRIFTS measurements.

*Spent catalysts; 8 h of time on stream (TOS).

**Figure 4:**
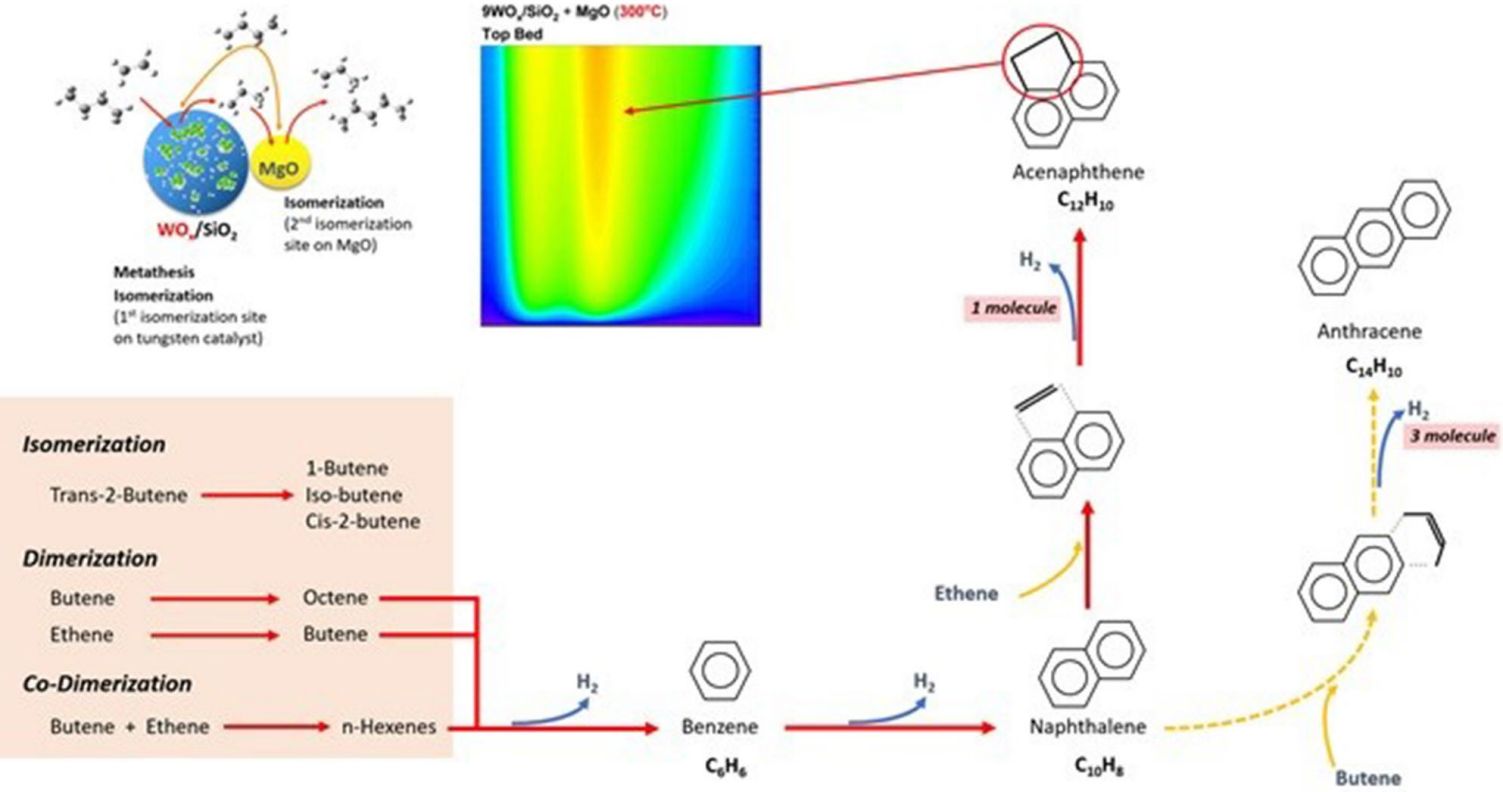
Schematic of possible reaction pathways at reaction temperature 300 °C. The proposed mechanism of hard coke generation from dimerization on WO_x_/SiO_2_ + MgO catalyst.

As described above, it can conclude that the increasing of tungsten metal loading affected the increasing of acidity that could improve the catalytic activity resulting in the increasing of coke formation. From the catalytic activity testing combining of *Operando* and in situ characterization techniques led to deep understanding the phenomena of coke formation on the WO_x_/SiO_2_ that physically mixed with MgO. The additional MgO not only enhanced the conversion of *trans*-2-butene because of isomerization reaction but also increased the amount of coke formation on the catalyst especially for soft coke formation. In this perspective, it should be proposed the occurrence of extremely hard coke that results of the decreasing role of metathesis under 320 °C due to the increasing of the dimerization to form the long chain of hydrocarbon and finally converted to the coke agglomeration on the surface of catalyst. So, increasing of adsorption ability of iso-butene and 1-butene with conjugated of ethene effect to generate the charged monoenyl or cyclopentenyl species eventually generated the extremely hard coke. Then those coke precursor molecules were easier to continue react with ethene because it’s necessary to remove one molecules of hydrogen when compared with butene that necessary to remove of three molecules (Fig. 4).

## Conclusions

Association of magnesium oxide with tungsten oxide silica supported catalyst effected to increase the isomer of butenes in the system. The reaction temperature at 450 °C of WO_x_/SiO_2_ catalyst was comprised with metathesis, isomerization and dimerization which were generated more products of C5^+^, resulting in the increasing the amount of coke deposited on catalysts. Addition of MgO physically mixed with WO_x_/SiO_2_ catalyst initiated the soft coke at temperature 260 °C when the reaction was tested 450 °C. However, reaction testing at 300 °C exhibited the extremely hard coke that could be the charged monoenyl or cyclopentenyl in the catalytic reaction system. It is necessary to burn out at temperature over 645 °C. It was noticed that when adding MgO physical mixed tungsten oxide silica supported catalyst, the operating temperature should be avoid in the range of 280–320 °C at atmospheric pressure due to the production of the new phenomena that generated the extremely hard coke and increased the quantities of coke deposited in the system resulting in the requirement of high temperature for catalyst regeneration and might need more frequent regeneration procedures as compared to the catalyst system at higher operating temperature. High temperature of regeneration effect to the cause of sintering of metal and maybe deformed the structure of catalyst. Therefore, it is necessary to study these phenomena to prevent the agglomeration of extremely hard coke and find an appropriate range of operating temperature.

## Abbreviations

DRIFTS: Diffuse reflectance infrared Fourier transform spectroscopy
XRD: X-ray powder diffraction
XPS: X-ray photoelectron spectroscopy
TPD: Temperature program desorption
GC-FID: Gas chromatograph-flame ionization detector
